# Supplementary material for: Chromosome-level genome assembly of Lilford’s wall lizard, Podarcis lilfordi (Günther, 1874) from the Balearic Islands (Spain)
Source: DNA Res. 2023 May 4;30(3):dsad008. doi: 10.1093/dnares/dsad008 (PMC10214862; doi:10.1093/dnares/dsad008)
Supplement: dsad008_suppl_Supplementary_Figure_S1 [file dsad008_suppl_supplementary_figure_s1.pdf]

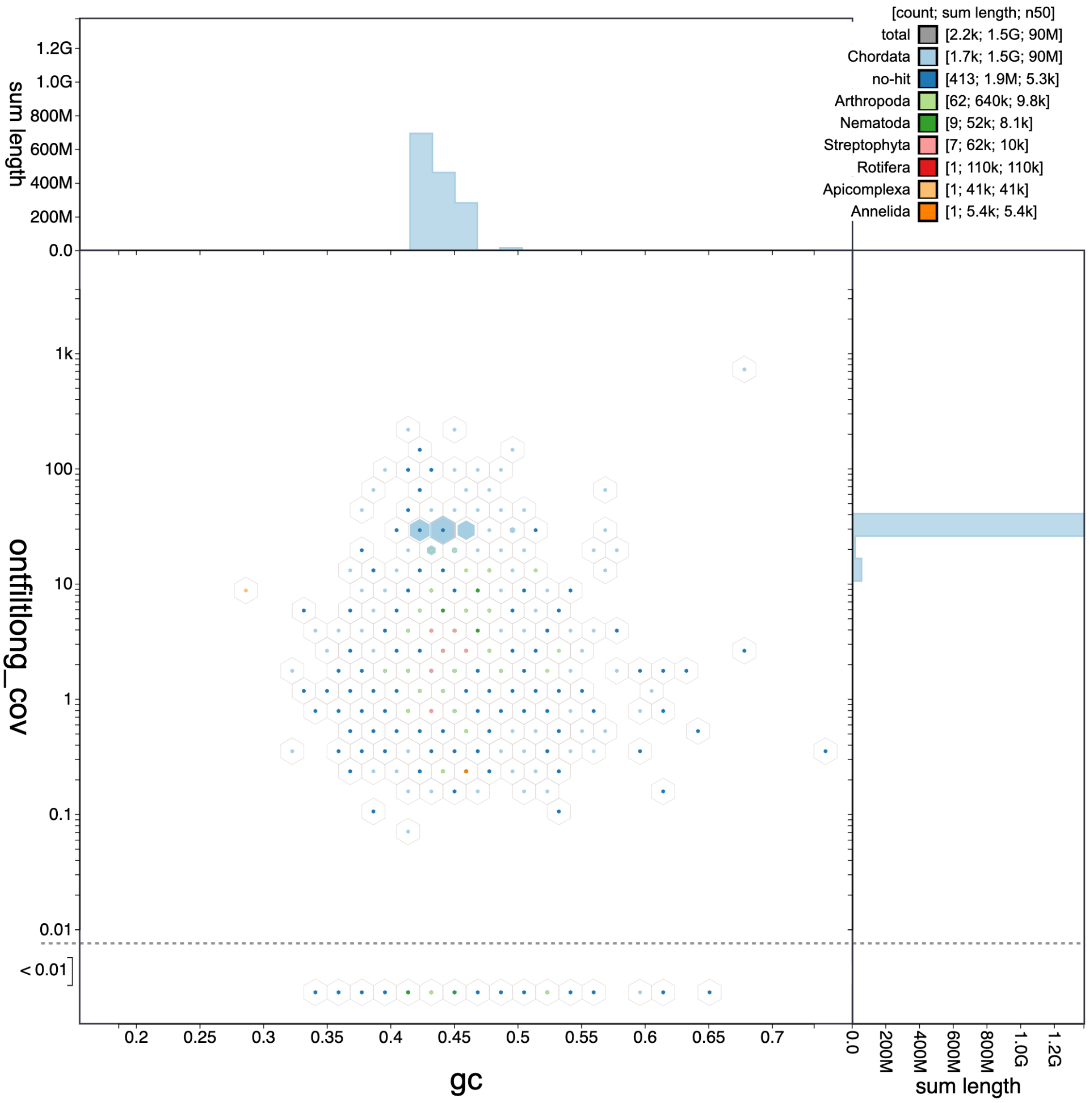

Figure S1: Hexagon-binned blob plot of base coverage of filtered ONT reads against GC proportion for scaffolds in assembly rPodLil1_1. Scaffolds are coloured by phylum (according to Blast hits against the nt database; last accessed September 2022) and binned at a resolution of 30 divisions on each axis. Coloured hexagons within each bin are sized in proportion to the sum of individual scaffold lengths on a square-root scale, ranging from 1,018 to 693,200,870. Histograms show the distribution of scaffold length sum along each axis.
