# Supplementary figures and images for: Chromosome-level genome assembly of Lilford’s wall lizard, Podarcis lilfordi (Günther, 1874) from the Balearic Islands (Spain)
Source: DNA Res. 2023 May 4;30(3):dsad008. doi: 10.1093/dnares/dsad008 (PMC10214862; doi:10.1093/dnares/dsad008)

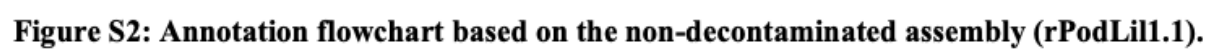

Supplement: dsad008_suppl_Supplementary_Figure_S2 [file dsad008_suppl_supplementary_figure_s2.pdf]
