# Supplementary material for: Chromosome-level genome assembly of Lilford’s wall lizard, Podarcis lilfordi (Günther, 1874) from the Balearic Islands (Spain)
Source: DNA Res. 2023 May 4;30(3):dsad008. doi: 10.1093/dnares/dsad008 (PMC10214862; doi:10.1093/dnares/dsad008)
Supplement: dsad008_suppl_Supplementary_Figure_S3 [file dsad008_suppl_supplementary_figure_s3.pdf]

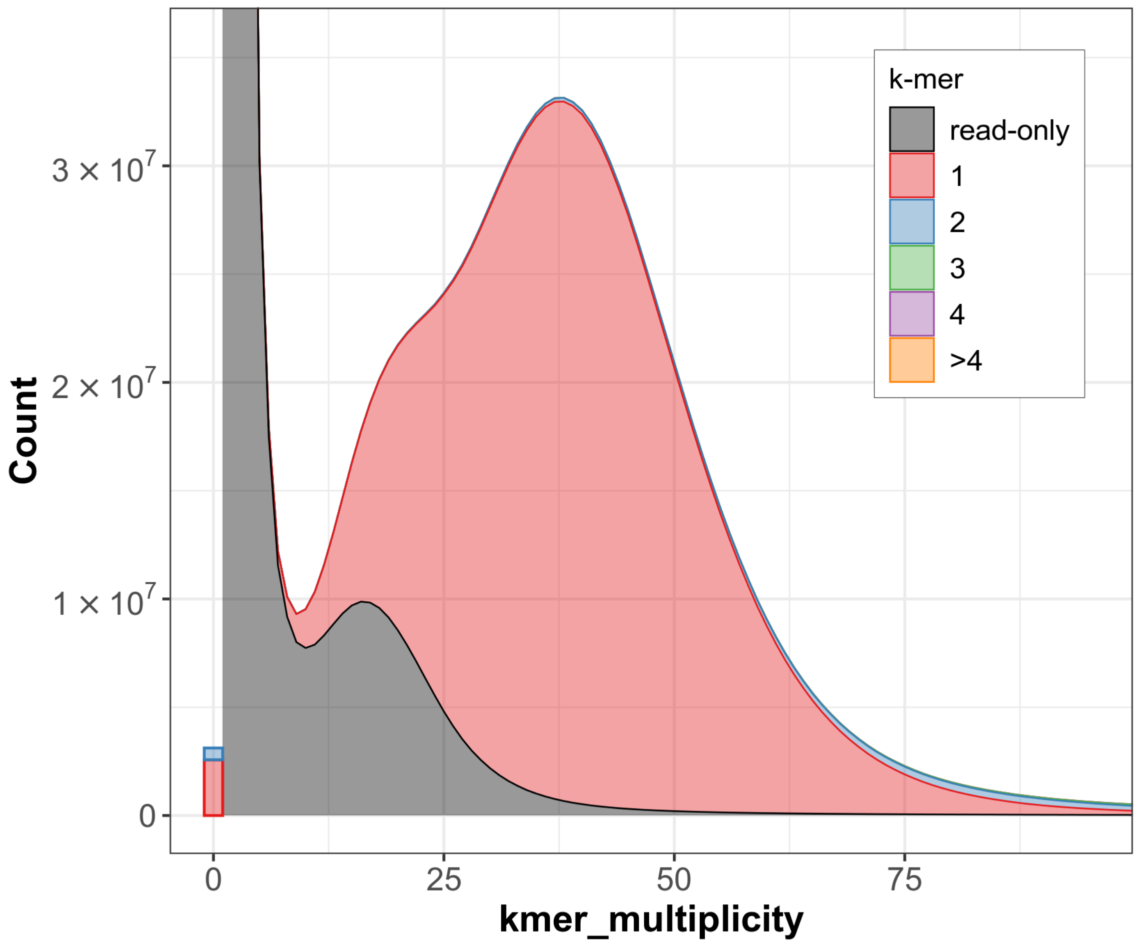

Figure S3: K-mer comparison between the Illumina reads and the rPodLil1.2 assembly. Stacked histogram of k-mer distributions obtained by comparing the assembly with Merqury v1.1 using k=21 on the 10X Illumina reads. Artificial duplications corresponding to duplicate k-mers are shown in blue above the main peak (~40x). They only account for 0.68% of the k-mers.
