# Supplementary material for: Chromosome-level genome assembly of Lilford’s wall lizard, Podarcis lilfordi (Günther, 1874) from the Balearic Islands (Spain)
Source: DNA Res. 2023 May 4;30(3):dsad008. doi: 10.1093/dnares/dsad008 (PMC10214862; doi:10.1093/dnares/dsad008)
Supplement: dsad008_suppl_Supplementary_Figure_S4 [file dsad008_suppl_supplementary_figure_s4.pdf]

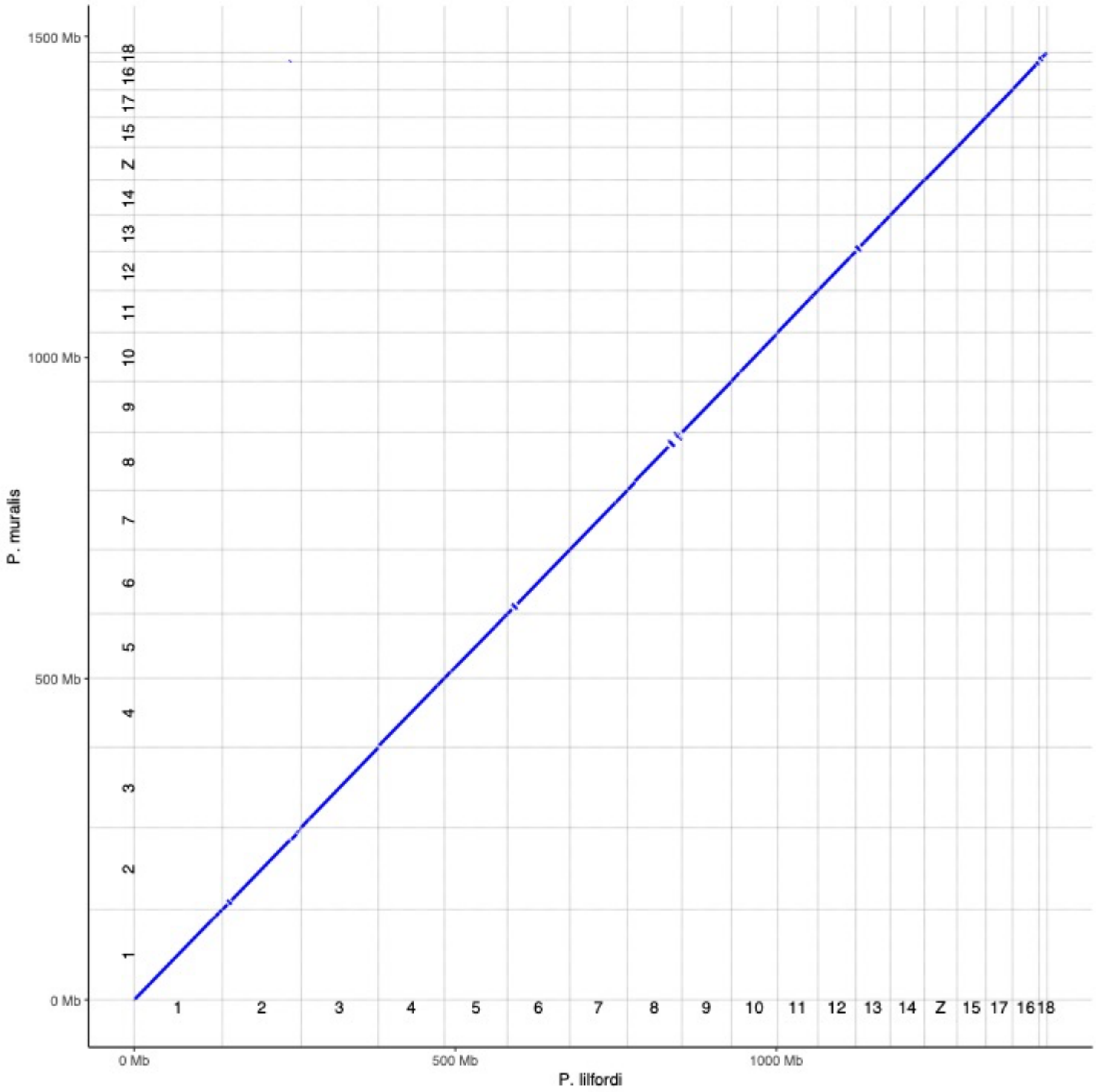

Figure S4: Whole genome alignment of *P. lilfordi* to *P. muralis*. Chromosomal sequences, named according to corresponding chromosomal sequences in *P. muralis*, are ordered from largest to smallest in *P. lilfordi* and oriented with respect to *P. muralis*, which inverts the order of chromosomes 16 and 17. Alignments longer than 100 kb were selected and visualized with the pafr R library.
