# Supplementary material for: Chromosome-level genome assembly of Lilford’s wall lizard, Podarcis lilfordi (Günther, 1874) from the Balearic Islands (Spain)
Source: DNA Res. 2023 May 4;30(3):dsad008. doi: 10.1093/dnares/dsad008 (PMC10214862; doi:10.1093/dnares/dsad008)
Supplement: dsad008_suppl_Supplementary_Figure_S5 [file dsad008_suppl_supplementary_figure_s5.pdf]

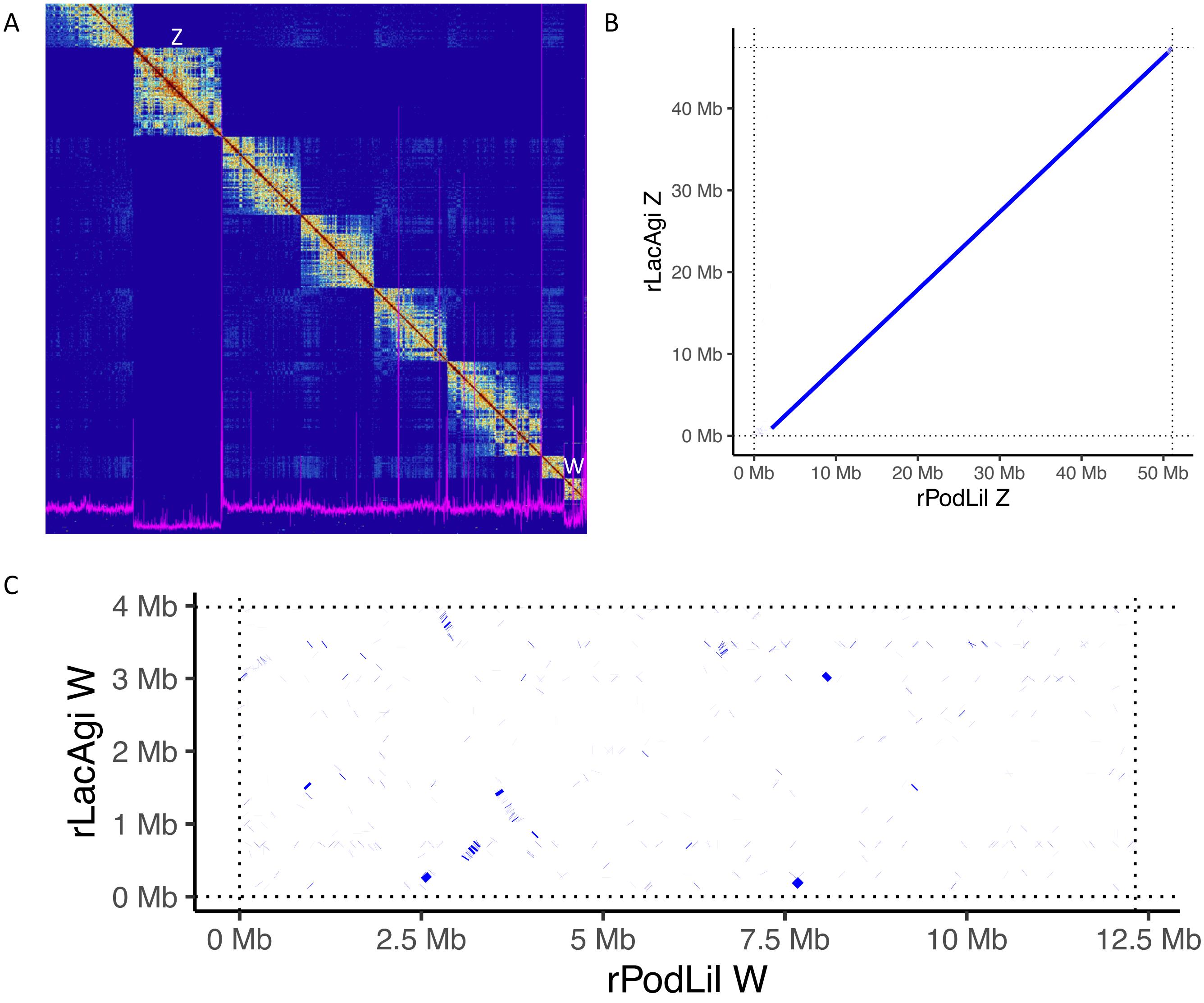

Figure S5: Sexual chromosome assignment. (A) Hi-C contact map showing scaffolds corresponding to the sexual chromosomes. Illumina coverage is plotted in pink. (B) Alignment of the scaffold assigned to the Z chromosome in *L. agilis* against the corresponding scaffold in *P. lilfordi*. (C) Alignment of the scaffold assigned to the W chromosome in *L. agilis* against the corresponding scaffold in *P. lilfordi*.
